# Differential Mechanisms Associated with Vascular Disrupting Action of Electrochemotherapy: Intravital Microscopy on the Level of Single Normal and Tumor Blood Vessels

**DOI:** 10.1371/journal.pone.0059557

**Published:** 2013-03-26

**Authors:** Bostjan Markelc, Gregor Sersa, Maja Cemazar

**Affiliations:** 1 Department of Experimental Oncology, Institute of Oncology Ljubljana, Ljubljana, Slovenia; 2 University of Primorska, Faculty of Health Sciences, Izola, Slovenia; University of California Berkeley, United States of America

## Abstract

Electropermeabilization/electroporation (EP) provides a tool for the introduction of molecules into cells and tissues. In electrochemotherapy (ECT), cytotoxic drugs are introduced into cells in tumors, and nucleic acids are introduced into cells in gene electrotransfer. The normal and tumor tissue blood flow modifying effects of EP and the vascular disrupting effect of ECT in tumors have already been determined. However, differential effects between normal vs. tumor vessels, to ensure safety in the clinical application of ECT, have not been determined yet. Therefore, the aim of our study was to determine the effects of EP and ECT with bleomycin on the HT-29 human colon carcinoma tumor model and its surrounding blood vessels. The response of blood vessels to EP and ECT was monitored in real time, directly at the single blood vessel level, by *in vivo* optical imaging in a dorsal window chamber in SCID mice with 70 kDa fluorescently labeled dextrans. The response of tumor blood vessels to EP and ECT started to differ within the first hour. Both therapies induced a vascular lock, decreased functional vascular density (FVD) and increased the diameter of functional blood vessels within the tumor. The effects were more pronounced for ECT, which destroyed the tumor blood vessels within 24 h. Although the vasculature surrounding the tumor was affected by EP and ECT, it remained functional. The study confirms the current model of tumor blood flow modifying effects of EP and provides conclusive evidence that ECT is a vascular disrupting therapy with a specific effect on the tumor blood vessels.

## Introduction

Electrochemotherapy (ECT) is a widely used treatment modality for the treatment of cancer, predominantly melanoma metastases in the skin. Its merits are increasingly exploited in other tumor types, and the technology is being adopted for the treatment of deep-seated tumors like liver, bone and brain metastases, and oesophageal and colon tumors [1], [2]. The basic principle of ECT exploits the physical phenomenon caused by electroporation/electropermeabilization (EP), where the application of electric pulses transiently permeabilizes the cell membrane, thus enabling the cell to admit those molecules which normally cannot enter it [3], [4]. In the case of ECT, the most commonly used drugs are bleomycin and cisplatin, where the application of EP to cells exposed to chemotherapeutic drugs potentiate their cytotoxic effect [1]. EP is also increasingly used for the delivery of different nucleic acids (plasmid DNA, siRNA etc.) into the cells *in vitro* and different tissues *in vivo*. This application of EP is termed gene electrotransfer (GET) and can be used in DNA vaccination [5], [6] or in the treatment of cancer [7]–[11].

The application of electric pulses to the tumors *in vivo* affects all cells in the tumor, particularly endothelial cells. Moreover, as a consequence, a pronounced tumor blood flow modifying effect was determined [12], [13]. Several studies on the blood flow modifying effects of EP and ECT on normal and tumor vasculature were performed using different indirect methods [13]–[21].

Recently, the effects of EP on normal skin blood vessels were thoroughly investigated by direct visualization. Namely, with the use of intravital microscopy in a mouse dorsal window chamber (DWC) model it was demonstrated that the application of electric pulses with different parameters leads to a rapid increase in skin blood vessel permeability for different sizes of molecules. Additionally, the application of electric pulses induced an immediate constriction of blood vessels, which was transient but still produced a reduction in the perfusion of the exposed vessels, the so-called vascular lock that lasted a maximum of 10 min [14], [16]. Furthermore, a histological analysis of the treated skin revealed extravasation of erythrocytes and infiltration of leukocytes together with some reversible damage to the skin [16]. These data provided a direct demonstration of the previously proposed mechanisms connected to the observed modification of the blood flow by application of electric pulses to normal tissues [15], [22]. However, whether the response of tumor vessels follows the same pattern as the response of normal vessels is unknown.

In tumors, the studies on vascular changes were conducted following both therapies, EP and ECT, using different indirect methods, including laser Doppler flowmetry, patent blue staining (PBV) and electron paramagnetic resonance. In these pre-clinical as well as clinical studies, ECT has been demonstrated to have, besides the blood flow modifying effect, also vascular disrupting action on tumors [13], [21]. In a clinical setting, this effect is mainly expressed as a cessation of bleeding of subcutaneous metastases, followed by crust formation [23]–[25]. In addition, it has been demonstrated that the blood flow modifying effects are longer-lived in tumors compared to normal tissues, and that the dose of chemotherapeutic drugs used in ECT predicts the extent of vascular disrupting action of ECT [22]. Furthemore, with electric pulses of higher energies (“irreversible electroporation – IRE”), a destruction of blood vessles is also observed, but it is limited to smaller vessels [25]–[27]. With the use of ECT and IRE for the treatment of cancer in well-perfused organs such as liver and brain, knowledge of the effects of ECT and EP on normal and tumor vasculature has become increasingly important [25], [26], [28]–[30]. Therefore, to gain an insight into the immediate and early effects of EP and ECT at the single tumor blood vessel level and to study the differential effect of EP and ECT on normal and tumor blood vessels, we used *in vivo* optical imaging of a human HT-29 colorectal carcinoma growing in DWC [31]–[33] in SCID mice, in combination with fluorescently labeled dextrans.

## Materials and Methods

### Reagents

The 70-kDa fluorescein isothiocyanate-labeled dextran (FD) (Sigma-Aldrich) and a 70-kDa Rhodamine-B-labeled dextran (RhD) were resuspended in phosphate-buffered saline (PBS). To remove any free fluorochromes or low molecular contaminants, the dextrans were washed two times for 3 h through 30-kDa Vivaspin ultrafiltration spin columns (Sartorius Stedim Biotech GmbH). The high molecular weight component was then resuspended in PBS to a final concentration of 37.5 mg/ml. Bleomycin (Pharmachemie B.V.) was resuspended in sterile distilled H_2_O to a working concentration of 3 mg/ml.

### Tumor Cells and Mice

HT-29 human colorectal adenocarcinoma cells (ATCC) were cultured in Advanced MEM (Gibco, Life Technologies) supplemented with 5% fetal bovine serum (Nalgene), 10 mM L-glutamine (Gibco), 50 µg/ml gentamicin (Krka) and 100 IU/l crystacillin (Pliva) in a 5% CO_2_ humidified incubator at 37°C. The cells were tested negative for mycoplasma using MycoFluor™ (Life Technologies). SCID mice (C.B-17/IcrHanHsd-Prkdc^scid^, Harlan) were held in a specific pathogen-free animal colony at controlled temperature and humidity with 12-h light/dark cycles. Food and water were provided *ad libitum*. Before the experiments, the mice were subjected to an adaptation period of 14 days. Experiments were performed on female mice, 12–14 weeks old and weighing 20–25 g. All animal experiments were conducted in accordance with the guidelines for animal experiments of the EU Directives and the permission obtained from the Ministry of Agriculture and the Environment of the Republic of Slovenia (Permission No. 34401-12/2009/6). For each experimental condition, 3–5 mice were randomly assigned. Only one experiment was performed on each mouse.

### Preparation of the Dorsal Window Chamber (DWC) and Tumor Induction in Mice

For DWC implantation, mice were first anesthetized with an intraperitoneal injection of ketamine (1 mg/ml, Narketan®, Vetoquinol AG), xylazine (5 mg/ml, Chanazine, Chanelle Pharmaceuticals Manufacturing Ltd.) and acepromazyne (0.4 mg/ml, Promace, Fort Dodge Animal Health). Then, the back of the mouse was depilated (Veet, Reckitt Benckiser Group). The DWC (APJ Trading Co.), consisting of two titanium frames, was surgically implanted onto the extended double layer of the skin, as described previously [14], [16], [33]. After the surgery and for the following 2 days, butorphanol (0.3 mg/kg, Torbugesic, Fort Dodge Animal Health) was injected intramuscularly. Tumors were induced 3 days after the surgery by injecting 5 µl of a dense cell suspension (10^8^ cells/ml of 0.9% NaCl) with a 29G needle into the dermis in the region of the DWC preparation, as described previously [34].

### Electropermeabilization (EP) and Electrochemotherapy (ECT)

**Figure 1:**
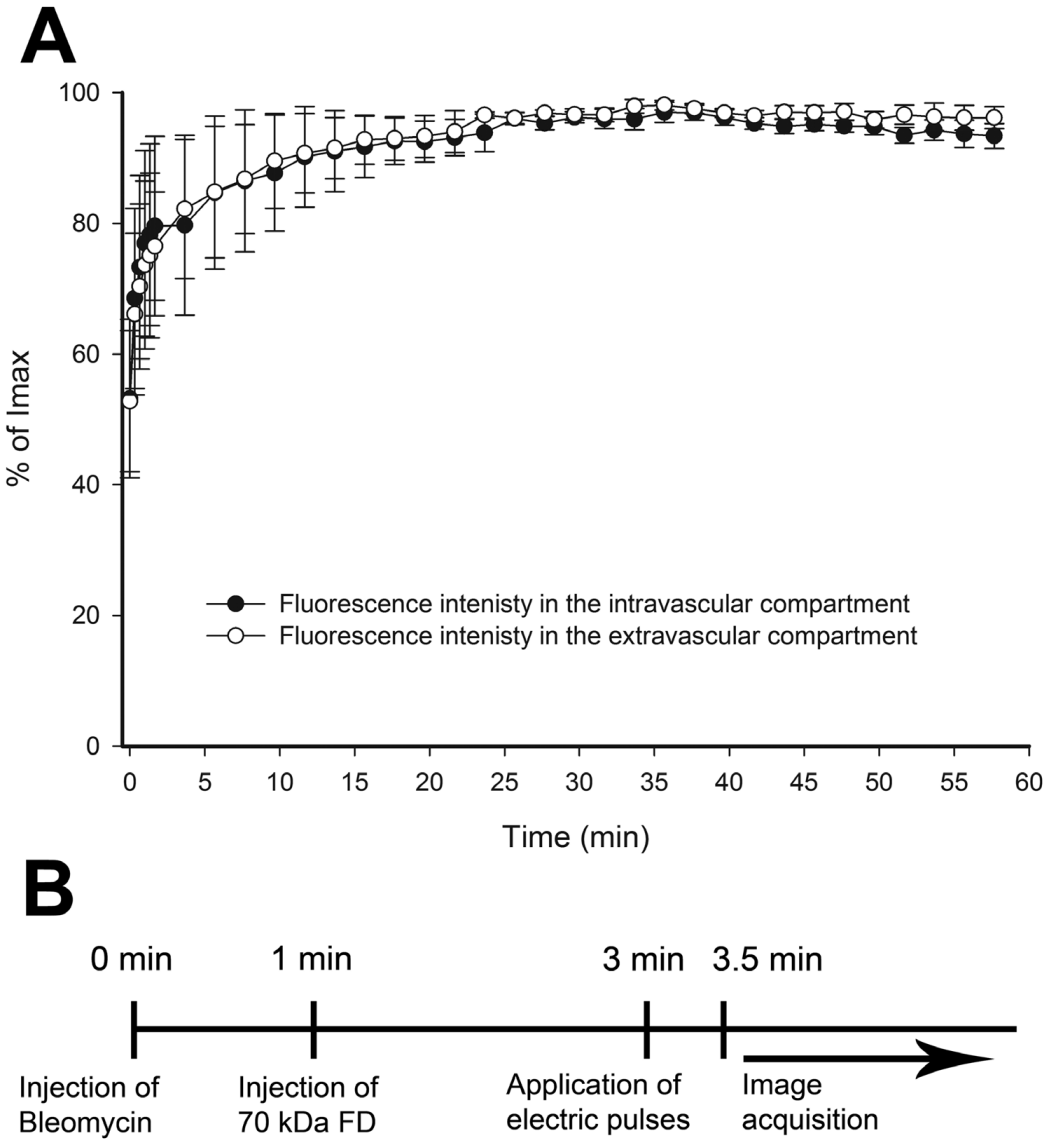
Characterization of the DWC model. (**A**) Filling of tumor blood vessels after *i.o.* injection of FD and increase of fluorescence intensity in the tumor tissue in control mice. Mean fluorescence intensities were expressed as a percentage of the maximum mean fluorescence intensity (Imax) reached in the observation period. The first image in the series was acquired ∼10 s after the *i.o.* injection of FD. (**B**) The timeline of the protocol used in the experiments.

EP or ECT was performed 6–10 days after the implantation, when the longest diameter of the tumors measured ∼2.5 mm. The pulsing parameters used were standard ECT parameters used in clinics [35]; 8 square-wave electric pulses with the voltage-to-distance ratio of 1300 V/cm, duration of 100 µs and frequency of 1 Hz. Pulses were generated by Cliniporator™ (IGEA S.r.l.) and delivered by two parallel stainless steel plate electrodes (30 mm long, 6 mm wide) placed 6 mm apart. The electrodes were placed on the skin on the opposite side of the cover glass, where the epidermis was intact, in a way so that the whole tumor and ∼1–2 mm of the surrounding normal tissue were encompassed between the electrodes. Good contact between the electrodes and the skin was ensured by means of a conductive gel (P. J. Dahlhausen & Co. GmbH). When ECT was performed, bleomycin (100 µg/mouse) was injected into the retro-orbital plexus (*i.o.*) 3 min before EP. To determine the extravasation of FD from the vessels, EP was performed 2 min after the *i.o.* injection of FD, when the vessels were ∼80% filled (Fig. 1A), or at different times before the FD *i.o.* injection to determine the duration of the “vascular lock”, the functional vascular density (FVD) and the average diameter of perfused tumor blood vessels (D_V_). The RhD-labeled dextran was injected *i.o.* only 4, 8 or 24 h after the therapy to visualize the perfused tumor blood vessels. A schematic of the protocol is presented in Fig. 1B.

### Intravital Microscopy and Image Acquisition

Intravital microscopy was carried out using a Carl Zeiss SteREO Lumar.V12 (Carl Zeiss) fluorescence stereomicroscope equipped with a NeoLumar S 0.8×objective (Carl Zeiss) and an MRc5 digital camera (Carl Zeiss). The animals were anesthetized by inhalation anesthesia (Isofluran, Nicholas Piramal India Ltd.) and placed on a custom-designed holder. Immediately (10 s) after the injection of 100 µl of FD or RhD, the image acquisition sequence was as follows: first, two identical series of images were taken, where images were acquired every 20 s for 2 min. In between both series, there was a 20-s pause when EP was performed, where applicable. Immediately afterwards, a third series of images was taken, where images were acquired every 2 min for 58 min. When FD or RhD were injected after EP, only the second and the third series of images were taken. The images were analyzed off-line with image analysis software (AxioVision, Carl Zeiss).

### Data Analysis

To determine the increase in fluorescence intensity inside and outside the vessels, indicating the extent of extravasation, image analysis was performed on the images of tumor blood vessels acquired at 80×magnification with a resolution of 1292×968 pixels and a 16-bit depth. Image analysis was performed as described previously [14], [16], with minor modifications: 1) on the aligned images, a region of interest (ROI) was determined from the entire field of view, which represented the tumor area; 2) the obtained mask was corrected, by hand with a graphic tablet (Genius, Taiwan), for the discrepancies between the mask and actual tumor blood vessels; 3), the corrected mask represented the tumor blood vessels network (intravascular space), and the inverted image of this mask represented the tumor tissue (extravascular space). After combining the masks with all of the aligned images of the acquired stacks, the variation in fluorescence intensity in the intravascular and extravascular spaces after EP or ECT was determined at all-time points.

To determine D_V_ and FVD, which describes the functional blood vessel density in a given ROI [36], the masks of the tumor blood vessels network were created on the image series, when FD was injected at different times after EP or ECT. The determined ROI represented the tumor area (A_T_), and the obtained masks of the tumor blood vessels network (intravascular space) represented the vascular area (A_V_). The tumor blood vessels in which FD was detected were considered perfused. From each created mask, vascular length (L_V_) was determined by measuring the length of the tumor blood vessels network skeleton. From the obtained parameters, FVD (FVD = L_V_/A_T_) and D_V_ (D_V_ = A_V_/L_V_) were calculated.

### Statistical Analysis

All data were tested for normality of distribution using the Shapiro-Wilk test. The differences between the experimental groups were evaluated by a Student’s t-test or one-way analysis of variance followed by a Holm-Sidak test for multiple comparisons. A p-value of less than 0.05 was considered to be statistically significant. SigmaPlot Software (Systat Software, Chicago, USA) was used for the statistical analysis and graphical representation.

## Results

### Vascular Pharmacokinetics and Extravasation of FD in Untreated Tumor Blood Vessels

For the maximal anti-tumor effect of ECT, the drug must be injected 3 min before EP [37], therefore the filling kinetics of the tumor blood vessels were determined after the injection of FD. The fluorescence of FD was detected already in the first acquired image after its *i.o.* injection (∼10 s) and reached ∼80% of its maximum intensity at 2 min post-injection in the tumor blood vessels as well as in the tumor tissue (Fig. 1A, Movie S1). After this initial rise, the fluorescence intensity remained constant during the observation period of 60 min (Fig. 1A). Therefore, subsequent experiments were performed according to the sequence presented in Fig. 1B.

### EP and ECT with Bleomycin Change the Extravasation Kinetics of FD from Tumor Blood Vessels

**Figure 2:**
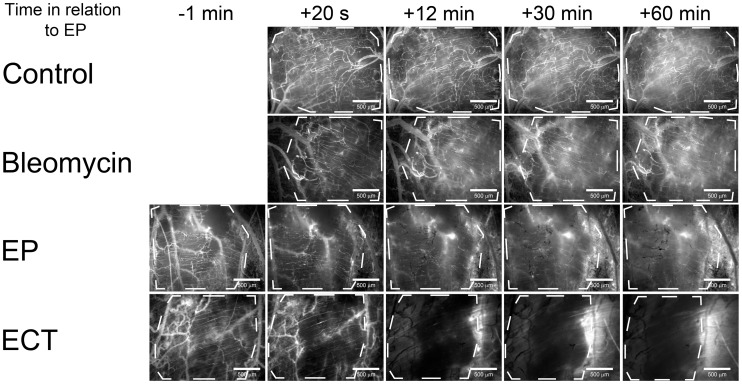
Imaging of the leakage of FD from tumor blood vessels into the tumor tissue. Tumor blood vessels were visualized by fluorescence microscopy at 80×magnification. Control – mice without treatment, Bleomycin – mice treated with bleomycin only, EP – mice treated with EP, ECT – mice treated with ECT. Tumors are marked with a dashed line. Scale bar is 500 µm.

The effect of EP and ECT on the permeability of tumor blood vessels was determined by relative variation of the mean fluorescence intensity in the tumor tissue due to the leakage of FD from the tumor blood vessels (Fig. 2).

**Figure 3:**
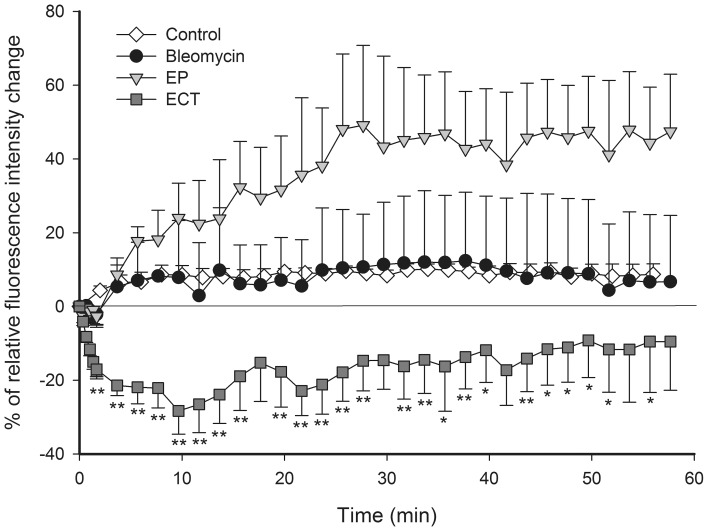
Quantification of FD leakage kinetics from tumor blood vessels. Relative mean fluorescence intensity changes as a function of time in the tumor tissue, outside the tumor blood vessels. Control – mice without treatment, Bleomycin – mice treated with bleomycin only, EP – mice treated with EP, ECT – mice treated with ECT. n = 3–5. **p<0.05 compared to all other groups, *p<0.05 compared to control and EP groups. Error bars indicate SEM.

When only EP was performed, the leakage of FD began with a short delay of 2 min and thereafter remained present throughout the observation period, but it was limited only to the tumor blood vessels, which were re-perfused after EP (Figs. 2, 3). In contrast, when ECT was performed, there was no evident leakage of FD from any tumor blood vessel (Fig. 2, Movie S2), and the mean fluorescence intensity in the tumor tissue statistically significant decreased (Fig. 3). However, leakage of FD from the normal vessels surrounding the tumor, which were also exposed to ECT, was extensive. The control mice and the mice treated with bleomycin only had no apparent dextran extravasation after the first few minutes (Figs. 1A, 3).

### EP and ECT Induce a “Vascular Lock”

EP and ECT both induced an abrogation of the blood flow, the so-called “vascular lock”. To determine the duration of the EP- and ECT-induced vascular lock, FD was injected at different times after EP or ECT.

**Figure 4:**
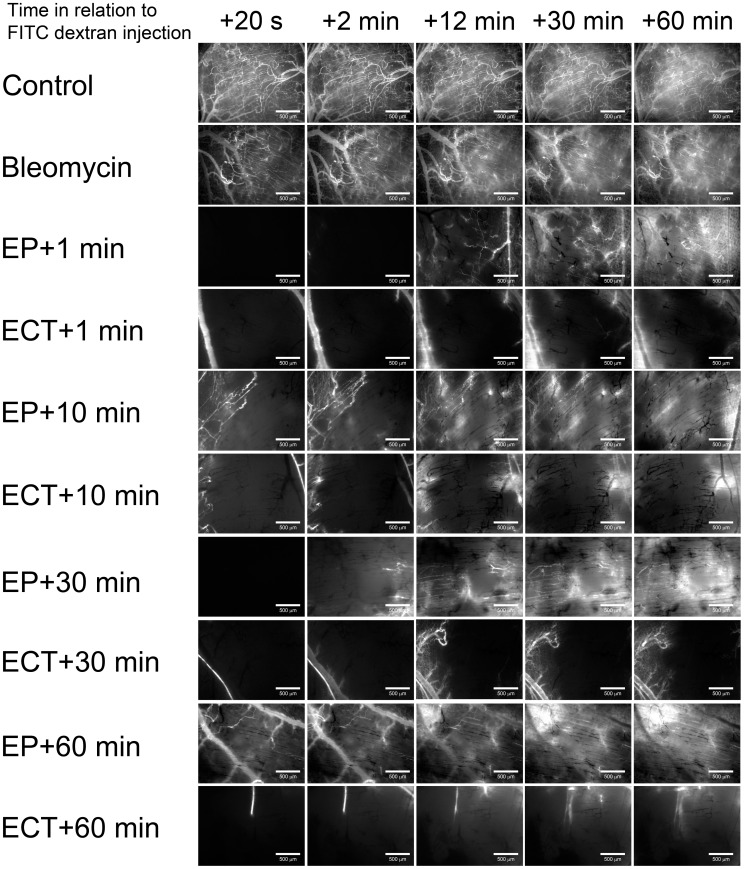
Illustration of a “vascular lock” and re-perfusion of tumor blood vessels after EP and ECT. Tumor blood vessels were visualized by fluorescence microscopy at 80×magnification. Control – mice without treatment, Bleomycin – mice treated with bleomycin only, EP – mice treated with EP, ECT – mice treated with ECT. (**A**) FD was injected *i.o.* at different times after the therapy (1, 10, 30, 60 min), and images were taken at designated times. Scale bar is 500 µm.

**Figure 5:**
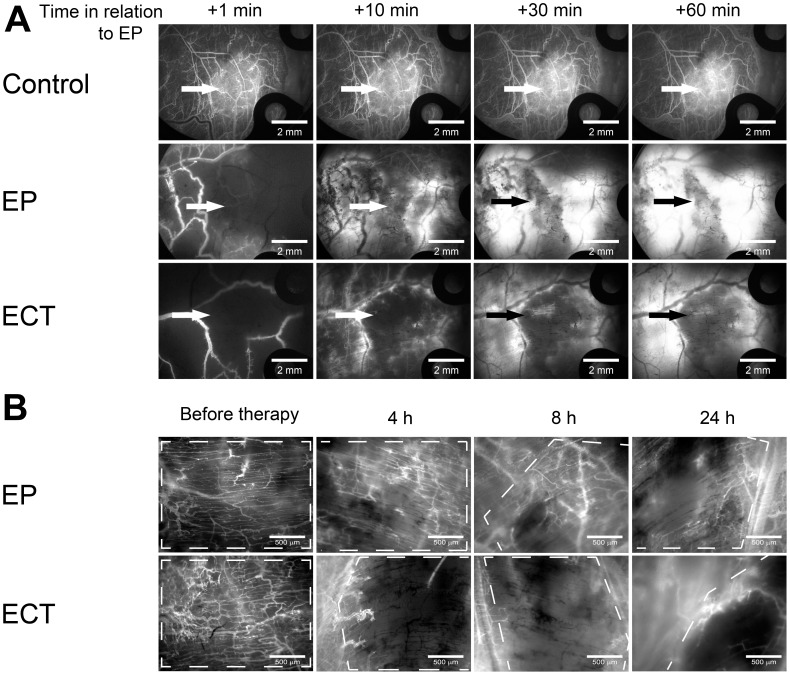
Illustration of a “vascular lock” and re-perfusion of tumor blood vessels in the first hour and 4–24 h after EP and ECT. Tumor blood vessels were visualized by fluorescence microscopy. Control – mice without treatment, EP – mice treated with EP, ECT – mice treated with ECT. (**A**) FD was injected *i.o.* 1 min after EP, and images were acquired at 20×magnification at designated times. Arrows indicate the position of the tumor. Scale bar is 2 mm. (**B**) RhD was injected *i.o.* at 4, 8 and 24 h after the therapy, and images were acquired 5 min after the injection at 80×magnification. The images are representative of different tumors. Tumors are marked with a dashed line. Scale bar is 500 µm.

When FD was injected 1 min after EP, the complete vascular lock lasted for ∼10 min, and a partial restoration of the blood flow was observed afterwards (Fig. 4, Movie S3). Extending the interval between EP and the injection of FD revealed that the tumor blood vessels started to slowly re-perfuse 10 min after EP (Fig. 4). However, not all vessels were perfused even at 24 h after EP (Fig. 5B). In contrast, when FD was injected 1 min after ECT, the vascular lock was virtually complete, and it lasted for the entire observation period of 60 min (Fig. 4). Extending the interval between ECT and the injection of FD to 60 min showed that, even 120 min after ECT, there were only a few tumor blood vessels at the periphery of the tumor that were perfused (Fig. 4, Movie S4). This remained almost unchanged for 24 h after ECT (Fig. 5B). In mice, where no EP was performed, the injected FD appeared in all tumor and surrounding blood vessels within 2 min after its injection (Figs. 4, 5A, movies S1, S5).

### EP and ECT Decrease Functional Vascular Density (FVD)

**Figure 6:**
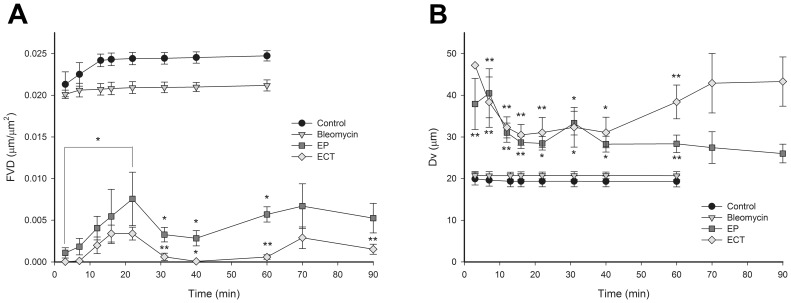
Timeline of the decrease in FVD and increase in D_V_ after EP and ECT. Control – mice without treatment, Bleomycin – mice treated with bleomycin only, EP – mice treated with EP, ECT – mice treated with ECT. (**A**) The changes in FVD within the tumors are presented as a function of time. (**B**) The changes of D_V_ within the tumors are presented as a function of time. n = 3–6. **p<0.05 compared to all other groups, *p<0.05 compared to control and bleomycin groups. Error bars indicate SEM.

FVD was determined to quantify the extent of the vascular lock after EP and ECT. Both therapies statistically significantly decreased the FVD throughout the entire observation period compared to the control and bleomycin groups (Fig. 6A). A small transient increase of the FVD was observed within 10 to 22 minutes after EP or ECT, indicating that re-perfusion of the tumor blood vessels has begun. However, 30 min after the therapy, the FVD decreased again, especially in the ECT group, where it was reduced to ∼0 µm µm^2^. In contrast, when only EP was performed, this decrease was less pronounced and lasted only 10 min. Thereafter, the FVD started to increase again.

### EP and ECT Differentially Affect Tumor Blood Vessels

In order to define whether EP and ECT affect a specific population of tumor blood vessels, a D_V_ was determined. The D_V_ of the control and bleomycin-treated tumors was ∼20 µm and did not change during the observation period. After the application of EP and ECT, the D_V_ increased immediately by 2-fold (Fig. 6B), which was statistically significantly larger compared to the D_V_ of the control and bleomycin groups. When ECT was applied, after an immediate increase of the D_V_, a small transient decrease in the D_V_ was observed between 10 and 40 minutes. However, afterwards, the D_V_ again increased to values above 40 µm. In contrast, when only EP was applied, the D_V_ started to decrease 10 minutes after the therapy and was approaching control values (Fig. 6B).

### EP and ECT Increase the Extravasation of FD from Normal Blood Vessels Surrounding the Tumor

**Figure 7:**
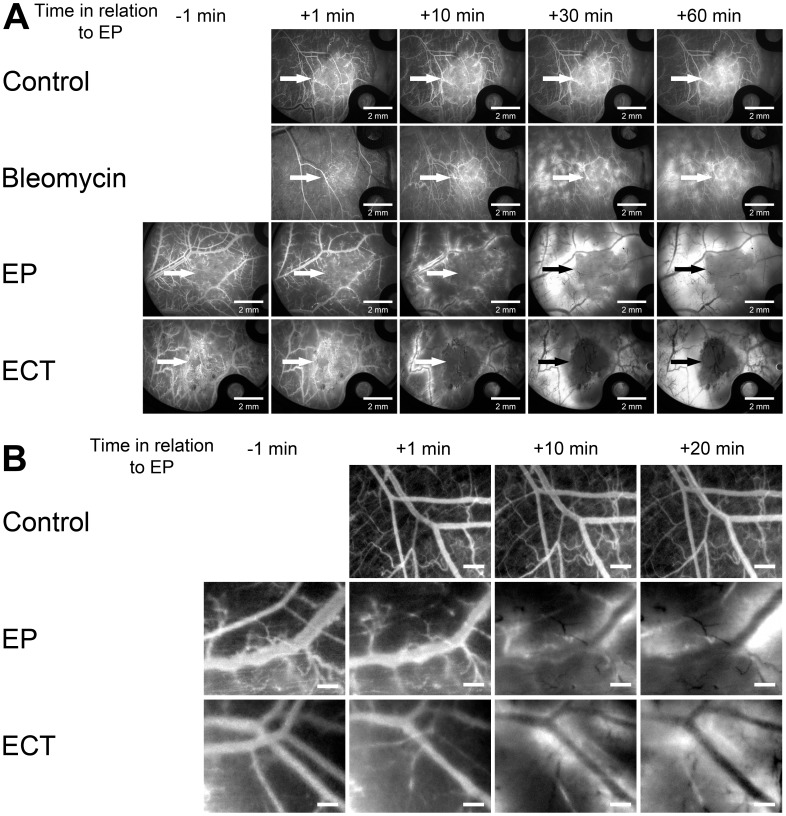
Illustration of FD leakage from blood vessels surrounding the tumor and their constriction after EP and ECT. Images were acquired at 20×magnification. Control – mice without treatment, Bleomycin – mice treated with bleomycin only, EP – mice treated with EP, ECT – mice treated with ECT. (**A**) Illustration of FD leakage from blood vessels surrounding the tumor, when FD was injected *i.o.* before the therapy. Arrows indicate the position of the tumor. Scale bar is 2 mm. (**B**) Illustration of the constriction of blood vessels surrounding the tumor visualized by FD. The enlarged sections of images were taken at 20×magnification. Scale bar is 500 µm.

To determine whether the normal blood vessels surrounding the tumors respond differently to EP and especially to ECT compared to the tumor blood vessels, images were acquired at 20×magnification every 10 minutes after the therapy. The application of electric pulses led to an immediate constriction of all exposed normal vessels (encompassed between the electrodes), increasing their permeability for FD (Fig. 7).

The constriction of all exposed vessels was localized to the area where electric pulses were applied, and it was transient (Fig. 7B). The vessels started to return to their pre-treatment size 10 min after EP. In the groups where no EP was applied, the size of the surrounding blood vessels did not change. The application of electric pulses led to increased leakage of FD, which was evident already 10 min after the therapy in the EP and ECT groups and lasted for the entire observation period of 60 min (Fig. 7A). The injection of bleomycin alone also increased the permeability of the surrounding blood vessels for FD. However, this effect was limited to small blood vessels only, and it was not localized. In the control group, there was no evident leakage of FD from the blood vessels surrounding the tumor (Fig. 7A, Movie S5). Furthermore, the vascular lock in the normal vessels surrounding the tumor followed the same pattern after ECT as after EP, demonstrating that ECT is not toxic for larger normal vessels (Fig. 5A, Movies S6, S7).

## Discussion

This study shows the *in vivo* response of tumor and normal blood vessels to EP and ECT observed at a single blood vessel level. The delivery of EP parameters, validated for the use in clinics [35], to tumors led to a vascular lock, decreased FVD and increased D_V_ within the tumor. In the case of ECT with bleomycin, the response of tumor blood vessels was more pronounced than after EP and resulted in the destruction of tumor blood vessels. Furthermore, ECT had no damaging effect on normal blood vessels surrounding the tumor, demonstrating that ECT has a specific vascular disrupting action only on the tumor blood vessels.

The primary cause of the anti-tumor effectiveness of ECT is increased cytotoxicity of the chemotherapeutic drug due to its increased uptake into the permeabilized cells [38]–[41]. However, besides direct cytotoxicity to tumor cells, ECT also has a vascular disrupting action [13], [22]. In addition, EP by itself has blood flow modifying effects in normal and tumor tissues [12]–[19], [22], [42]. The current model describing the blood flow modifying effects of EP and the vascular disrupting action of ECT in tumors is based on physiological data obtained by indirect methods [22]. Our study is the first to provide a direct, detailed observation of the effects of EP and ECT on the tumor blood vessels and the surrounding vasculature at the single blood vessel level.

The filing kinetics of the tumor blood vessels with FD (Fig. 1A), which was chosen because of its pharmacokinetics properties [31], confirmed that the standard ECT protocol can be used in a DWC [37]. Thus, our experimental setting allowed us to observe the vascular effects in real time. The observed blood flow abrogation – the vascular lock – in the tumors after EP and ECT (Fig. 4) is in accordance with the proposed model and the studies performed using laser Doppler flowmetry and PBV [13], [21]. We confirmed that the onset of a vascular lock is immediately after the application of electric pulses, and that it affects the entire tumor vasculature (Fig. 4, Movies S3, S4). This was accompanied by a reduction of FVD in the tumor (Fig. 6A) as well as by an increase of the D_V_ of the tumor blood vessels (Fig. 6B), which has not been demonstrated before. In normal blood vessels, the vascular lock effect lasts ∼8 min and is attributed to the sympathetically mediated vasoconstriction of arterioles due to the effect of EP on the smooth muscle cells and interstitial edema resulting from the leakage of proteins from the permeabilized cells in the tissue exposed to the electric pulses in combination with reduced intravascular pressure because of the permeabilization of blood vessels wall [14]–[16]. However, the lack of smooth muscle cells in the tumor blood vessels implies that the same principle cannot be applied to tumor vasculature. It was proposed that the first phase of the vascular response to EP and ECT is due to a direct vasoconstrictive effect on the tumor-supplying arterioles, which is the main mechanism of action of some chemical vascular disrupting agents (VDAs) [43]. Our data confirmed that the tumor-supplying arterioles respond to the application of electric pulses in the same way as the normal vessels, with rapid vasoconstriction and increased permeability (Figs. 5A, 7A, Movies S6, S7) [14], [16], which is therefore the main cause of the immediate vascular lock observed after EP and ECT of tumors. Furthermore, the blood flow in the blood vessels surrounding the tumor started to return to normal level ∼10 min after EP or ECT (Fig. 5A, 7A), which is in agreement with our previous results [14], [16]. This resulted in a transient re-perfusion of several vessels in the tumor, especially in its rim, giving further evidence that the first phase of the EP-mediated tumor blood flow modifying effects can be attributed to vasoconstriction of the tumor-supplying arterioles. The transient re-perfusion of the tumor blood vessels was accompanied by an increase in the FVD and a decrease in the D_V_ that was present between 10 and 30 min after the therapy (Fig. 6), confirming the results obtained by laser Doppler flowmetry, where the blood flow returned to 40% of the values observed before EP or ECT within 15 min after the therapy [13], [22].

A second decrease in the FVD was determined 30 min after the therapy in both groups where EP was applied (Fig. 6A), which is in accordance with the experimental data obtained by laser Doppler flowmetry [13] and the second phase of the proposed model. This was attributed to a profound disruption of the cellular cytoskeleton after EP and to an increase in the HUVEC endothelial cell monolayer permeability [22], [44]. These changes were reversible, and all cytoskeletal structures were recovered within 1–2 h after EP [44]. In *in vivo* settings, the observed changes may lead to an increased resistance to blood flow and to an increased leakage of proteins from the blood vessels, thus increasing the interstitial fluid pressure, decreasing the intravascular pressure and causing interstitial edema [22], [44]. Our data on the measurement of FD leakage show that EP increases the permeability of the tumor blood vessels (Figs. 2, 3), confirming the *in vitro* data on the HUVEC monolayer permeability and the results for normal blood vessels *in vivo* [14], [16], [44]. The increased permeability of the tumor blood vessels after EP was probably underestimated due to the vascular lock and, consequently, the lack of FD in the tumor blood vessels after EP. However, the increased leakage of FD could still be detected due to the re-perfused vessels after EP (Fig. 3). When ECT was applied, no increase of FD was observed. Moreover, after the therapy, a decrease in the fluorescence intensity in the tumor tissue was determined (Fig. 3). Although the results suggest a different effect of ECT on the permeability of tumor blood vessels compared to EP, the difference is probably only due to a more profound vascular lock effect when ECT was applied as explained below.

The previously proposed model suggests that the response of the tumor blood vessels to EP and ECT starts to differ when the cytotoxic effect of ECT destroys the endothelial cells approximately 2–8 h after the therapy [22]. However, our data show that the differences occur already within the first hour after the therapy. Firstly, ECT induces a more profound vascular lock (Fig. 2). Secondly, 40 minutes after the therapy, when re-perfusion begins in the tumors treated only with EP, probably due to the normalization of cytoskeletal organization and re-establishment of cell-to-cell junctions in tumor endothelial cells [44], but there is still no re-perfusion in tumors treated with ECT. Moreover, a further decrease in the FVD and increase of the D_V_ were determined, indicating that the effects of bleomycin in tumor endothelial cells are present already at 40 min post-ECT. The differences between EP and ECT become even more evident 4 h post EP, when a partial restoration of blood flow in the tumors treated with EP only was determined, whereas in the tumors treated with ECT, there was no blood flow detected even 24 h after the therapy (Fig. 5B), indicating that the cytotoxic effect of ECT on tumor endothelial cells [45] destroys the tumor blood vessels, which may lead to ischemic death of the cells supplied by the affected blood vessels [22]. Of utmost importance is also the fact that the normal blood vessels surrounding the tumor were not destroyed after ECT and retained their functionality after the therapy (Figs. 5A, 7A, Movies S6, S7). In clinical settings, this phenomenon was also observed as differential effect on tumor and normal tissues in the terms of toxicity and in even larger normal vessels surrounding tumor that were not damaged after ECT [29], [35], [46]. Namely, tumor lying in-between large blood vessels in the liver was affected by electrochemotherapy, while large normal blood vessels in liver, close to the tumor, remained viable after ECT [29]. The underlying mechanisms for the observed differential effect on the tumor and normal blood vessels of ECT are unknown but might be due to the difference in the normal and tumor endothelial cells, as the latter proliferate at a much higher rate and are therefore more susceptible for apoptosis [47], [48]. In addition, so far, the experimental *in vitro* studies of the EP and ECT effects on endothelial cells were performed only on normal endothelial cells, therefore they may not adequately represent the situation in tumor endothelial cells, which have a much less stable cytoskeletal organization, making them more susceptible to damage induced by the disruption of their cytoskeleton [44], [45], [49]–[51].

Vascular effects were observed also after IRE, where small tumor blood vessels were reported to be destroyed within 24 h, whereas the larger ones remained functional [27], [52]. In another study, different energies of electric pulses, also similar to those used in ECT, were tested directly on the normal artery. The authors demonstrated by histological analysis performed 7 days after the treatment no long-term damage to the endothelial cell lining of the large artery, as well as to its vascular smooth muscle cells (VSMC) [25], [26], [53]. However, with increasing the number and amplitude of electric pulses, the endothelial cell lining of large arteries and the VSMC were destroyed, but the scaffold of the blood vessels remained functional allowing the repopulation of the endothelial layer with the endothelial cells is following over a few weeks’ time [25], [26], [53], [54]. Taken together, our results and that of IRE studies demonstrate, although using different endpoints, that small tumor blood vessels are more susceptible to EP, than larger and normal vessels. These findings further support the safety of EP and ECT for the use in the well perfused organs, such as liver and brain, where vascular disrupting action is exerted on small tumor blood vessels, however the preserved functionality of the large normal blood vessels is crucial for the recovery of the treated tissue.

In conclusion, to our best knowledge, this is the first study that shows the differential effects of EP and ECT on tumor and normal blood vessels directly at a single blood vessel level in real time. The results of our study confirm the proposed two-phase model of the blood flow modifying effects of EP and ECT on tumor blood vessels but also demonstrate for the first time that the response of the tumor blood vessels to ECT starts to differ from the response to EP already within the first hour after the therapy. Furthermore, it provides additional evidence that ECT has a selective vascular disrupting action on tumors by destructing small tumor blood vessels, without affecting the larger normal blood vessels surrounding the tumor. Thus, with the focus of clinical ECT shifting to well-perfused internal organs, such as liver and brain [28]–[30], our data provide strong evidence for a safe implementation of ECT in them.

## Supporting Information

Movie S1 **The filing kinetics of the tumor blood vessels after** ***i.o.*** **injection of FD in control mice.** Tumor blood vessels were visualized by fluorescence microscopy at 80×magnification. Scale bar 500 µm. Time in the movie is expressed in relation to FD injection.
(AVI)

Movie S2 **Imaging of perfusion changes of tumor blood vessels and leakage of FD after ECT.** Bleomycin (100 µg/mouse) was injected *i.o.* 3 min before EP (8 square wave electric pulses, voltage-to-distance ratio 1300 V/cm, duration 100 µs, repetition frequency 1 Hz) and the FD was injected *i.o.* 1 min after EP. Tumor blood vessels were visualized by fluorescence microscopy at 80×magnification. Scale bar 500 µm. Time in the movie is expressed in relation to EP.
(AVI)

Movie S3 **Imaging of the induced vascular lock and subsequent re-perfusion of tumor blood vessels after EP.** FD was injected *i.o.* 1 min after the application of electric pulses (8 square wave electric pulses, voltage-to-distance ratio 1300 V/cm, duration 100 µs, repetition frequency 1 Hz). Tumor blood vessels were visualized by fluorescence microscopy at 80×magnification. Scale bar 500 µm. Time in the movie is expressed in relation to EP.
(AVI)

Movie S4 **Imaging of the induced vascular lock and subsequent re-perfusion of tumor blood vessels after ECT**. Bleomycin (100 µg/mouse) was injected *i.o.* 3 min before EP (8 square wave electric pulses, voltage-to-distance ratio 1300 V/cm, duration 100 µs, repetition frequency 1 Hz) and the FD was injected *i.o.* 1 min after EP. Tumor blood vessels were visualized by fluorescence microscopy at 80×magnification. Scale bar 500 µm. Time in the movie is expressed in relation to EP.
(AVI)

Movie S5 **The filing kinetics of the blood vessels surrounding the tumor after** ***i.o.*** **injection of FD in control mice.** Blood vessels were visualized by fluorescence microscopy at 20×magnification. Scale bar 2 mm. Time in the movie is expressed in relation to FD injection.
(AVI)

Movie S6 **Imaging of the induced vascular lock and subsequent re-perfusion of blood vessels surrounding the tumor after EP.** FD was injected *i.o.* 1 min after the application of electric pulses (8 square wave electric pulses, voltage-to-distance ratio 1300 V/cm, duration 100 µs, repetition frequency 1 Hz). Blood vessels were visualized by fluorescence microscopy at 20×magnification. Scale bar 2 mm. Time in the movie is expressed in relation to EP.
(AVI)

Movie S7 **Imaging of the induced vascular lock and subsequent re-perfusion of blood vessels surrounding the tumor after ECT.** Bleomycin (100 µg/mouse) was injected *i.o.* 3 min before EP (8 square wave electric pulses, voltage-to-distance ratio 1300 V/cm, duration 100 µs, repetition frequency 1 Hz) and the FD was injected *i.o.* 1 min after EP. Blood vessels were visualized by fluorescence microscopy at 20×magnification. Scale bar 2 mm. Time in the movie is expressed in relation to EP.
(AVI)
